# Vanadium and tantalum doping of tin dioxide: a theoretical study

**DOI:** 10.1038/s41598-023-47383-3

**Published:** 2023-11-28

**Authors:** Petros-Panagis Filippatos, Nikolaos Kelaidis, Maria Vasilopoulou, Alexander Chroneos

**Affiliations:** 1 Institute of Nanoscience and Nanotechnology (INN), National Center for Scientific Research Demokritos, Agia Paraskevi, 15310 Athens, Greece; 2 Faculty of Engineering, Environment and Computing, Coventry University, Priory Street, Coventry, CV1 5FB UK; 3 Theoretical and Physical Chemistry Institute, National Hellenic Research Foundation, Vass. Constantinou 48, 11635 Athens, Greece; 4 Department of Materials, Imperial College, London, SW7 2AZ UK; 5 Department of Electrical and Computer Engineering, University of Thessaly, 38221 Volos, Greece

**Keywords:** Materials for energy and catalysis, Theory and computation

## Abstract

The increasing demand of efficient optoelectronic devices such as photovoltaics has created a great research interest in methods to manipulate the electronic and optical properties of all the layers of the device. Tin dioxide (SnO_2_), due to his charge transport capability, high stability and easy fabrication is the main electron transport layer in modern photovoltaics which have achieved a record efficiency. While the wide band gap of SnO_2_ makes it an effective electron transport layer, its potential for other energy applications such as photocatalysis is limited. To further improve is conductivity and reduce its bandgap, doping or co-doping with various elements has been proposed. In the present density functional theory (DFT) study, we focus on the investigation of vanadium (V) and tantalum (Ta) doped SnO_2_ both in the bulk and the surface. Here we focus on interstitial and substitutional doping aiming to leverage these modifications to enhance the density of states for energy application. These changes also have the potential to influence the optical properties of the material, such as absorption, and make SnO_2_ more versatile for photovoltaic and photocatalytic applications. The calculations show the formation of gap states near the band edges which are beneficial for the electron transition and in the case of Ta doping the lowest bandgap value is achieved. Interestingly, in the case of Ta interstitial, deep trap states are formed which depending of the application could be advantageous. Regarding the optical properties, we found that V doping significantly increases the refractive index of SnO_2_ while the absorption is generally improved in all the cases. Lastly, we investigate the electronic properties of the (110) surface of SnO_2_, and we discuss possible other applications due to surface doping. The present work highlights the importance of V and Ta doping for energy applications and sensor applications.

## Introduction

SnO_2_ also known as cassiterite and stannic oxide, represents one of the most used wide bandgap semiconductors in energy devices^1,2^. It is characterized by n-type conductivity, which can be attributed to its intrinsic defects, such as oxygen vacancies^3^ and tin interstitials^4^. As a compound, SnO_2_ exhibits low resistivity^5^ and high-dielectric constant, and has therefore been considered for gate oxides on Si-based electronic devices^6,7^ as well as for electron transport layer in perovskite photovoltaics^8^. Undoped SnO_2_ has a wide bandgap value of ~ 3.6 eV^9^ and its reported resistivity values range from 10^−2^ to 10^−3^ Ω cm^10^. One of the main quests in photovoltaic technologies is to enhance the conductivity of the electron transport layer without reducing the bandgap^11^. In essence, the efficiency of the devices is highly connected with the charge recombination and losses due to the limited transport properties of the used layers. Furthermore, the stoichiometric SnO_2_ exhibits low intrinsic carrier density and low mobility of its charges due to the oxygen vacancies which act as donors. Doping is examined as a strategy to further reduce the resistance of SnO_2_ and to enhance the transition in the visible wavelengths^12,13^. For photocatalysis, it is important to increase the conductivity of the photocatalyst and decrease the bandgap of the semiconductor. The increase in the carrier concentration makes the intermediate energy gap between valence band and conduction band more active^14^.

To further improve the response of gas sensing devices and improve the sensitivity and selectivity, it is common to add porous materials and catalytically active agents^15,16^. Doping the metal oxide -such as SnO_2_ – based sensor promotes the physicochemical reactions between the surface and the gas^17^. Various reports propose that incorporation of the appropriate dopant is an efficient technique to enhance the sensitivity, selectivity, operating temperature and recovery time of SnO_2_ based gas sensor as the dopant modifies the structural, electronic and optical properties of the host compound^18,19^. Transition metal elements such as Zn, Mn, Cr etc. have been proposed by researchers as successful dopants which enhance the response and selectivity of SnO_2_^20–23^. Although various studies^24–26^ have explored the electrical and optical properties of Ta doped SnO_2_, the applications of this doping method in gas sensor has not be reported or examined. Interestingly, Liu et al*.*^27^ developed a Ta:SnO_2_ electron transport layer (ETL) for high efficiency perovskite solar cells. Ben Soltan et al*.*^28^ developed V:SnO_2_ nanoparticles for photocatalytic applications.

Here we employ hybrid functional DFT to investigate the impact of substitutional and interstitial doped V and Ta doped bulk and (110) surface SnO_2_.

## Methodology

The Cambridge Serial Total Energy Package (CASTEP)^29^ was used. To encounter the effect of localized electrons and bandgap underestimation we employed the hybrid functional PBE0 with norm conserving pseudopotentials^30^. Convergence tests revealed that a cutoff energy was chosen of 800 eV and 2 × 2 × 3 k-points for the sampling of the Brillouin zone were sufficient for the 48 atom supercell (2 × 2 × 2 unit cells). The supercell was chosen by taking into account that although hybrid functionals can provide more reliable results, they are very computationally expensive. For the optimization of the relaxed structures and the prediction of the ground state of each system, we used the Broyden–Fletcher–Goldfarb–Shanno (BFGS) method which was seen to predict the correct ground state to various different system^31^. For the interstitial positions we examined all possible configurations in the supercell in conjunction with geometry optimization and retained the lowest energy configuration. Specifically, we placed the interstitial defects in various sites and we used as the final ground state the configuration with the minimum total energy. The surface simulation was based on a slab model with a vacuum of about 12 Å vertical to the (110) direction. Here, the top two layers represent the surface, whereas the bottom two layers are fixed and represent the bulk region. Considering the DOS calculations a k-point mesh of 5 × 5 × 5 was used for bulk and 3 × 3 × 1 set for the surface. We set the convergence criteria at 2.0 × 10^−5^ eV/atom for the SCF tolerance, 0.05 eV/Å for the force tolerance and 0.001 Å for the max displacement tolerance.

## Results and discussion

### Bulk SnO_2_

**Figure 1:**
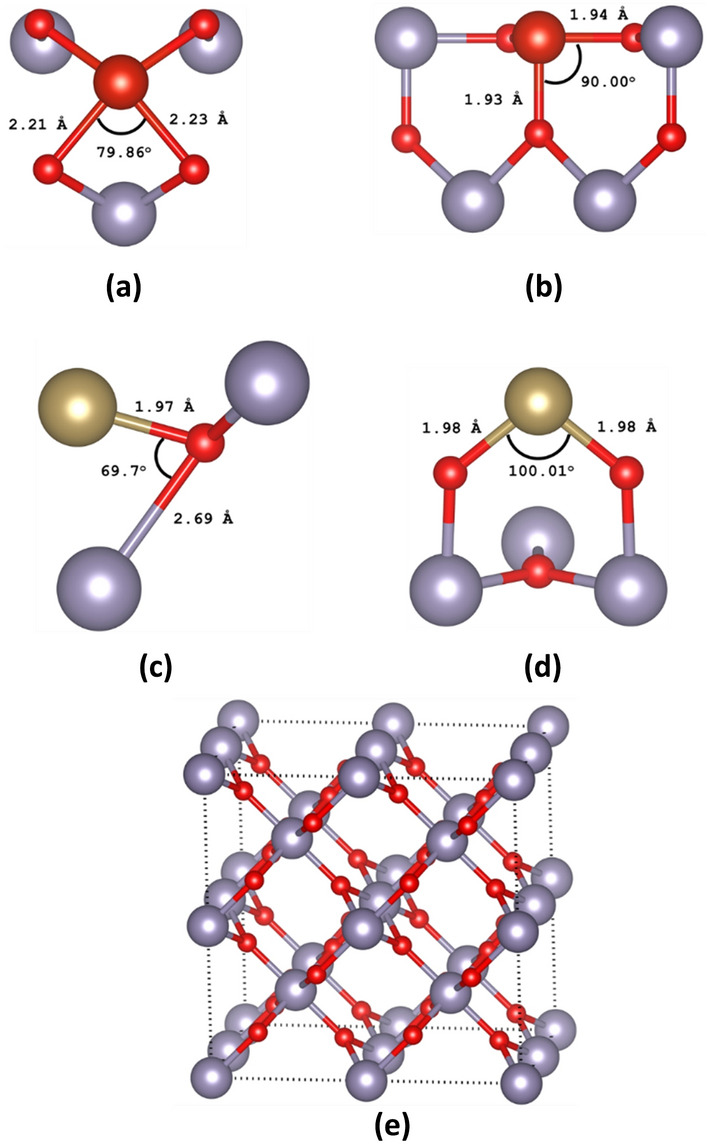
The minimum energy configurations of (**a**) Ta substitutional doped SnO_2_ (Ta_Sn_:SnO_2_), (**b**) Ta interstitial doped SnO_2_ (Ta_i_:SnO_2_), (**c**) V substitutional doped SnO_2_ (V_Sn_:SnO_2_), (**d**) V interstitial doped SnO_2_ (V_i_:SnO_2_) and (**e**) the supercell.

Here we considered SnO_2_ in the rutile structure (space group number P4_2_/mmm), with determined unit cell parameters a = b = 4.737 Å and c = 3.186 Å and our DFT calculated unit cell parameters are a = b = 4.717 Å and c = 3.189 Å^14^. In Fig. 1, we report the minimum energy configurations with relevant nearest neighbour distances and angles of (a) Ta substitutional doped SnO_2_ (Ta_Sn_:SnO_2_), (b) Ta interstitial doped SnO_2_ (Ta_i_:SnO_2_), (c) V substitutional doped SnO_2_ (V_Sn_:SnO_2_), (d) V interstitial doped SnO_2_ (V_i_:SnO_2_) and (e) the undoped 48-atom supercell. We have considered all the possible configurations of these defects in the supercell but the subsequent figures report results on the minimum energy configurations only.

In Table 1 we have gathered the lattice parameters for all the doping cases. As it is seen, in all the cases except the vanadium substitutional, the volume of the unit cell increases. Typically, a larger supercell is connected to larger area for the chemical reactions to take place and can lead to improved photocatalytic activity of SnO_2_. Our approach agrees well with experimental reports^23,32^. Specifically, Alvarez-Roca et al.^23^ investigated at various vanadium doping concentration the X-ray diffraction and transmittance electron microscope for the determination of the structural changes in SnO_2_. Similar to our study, the found that the V atoms can be incorporated inside the structure of SnO_2_ and at low concentrations the cell volume is reduced. According to their study, this reduction enhances the specific surface area to volume ration which is highly beneficial for applications of V:SnO_2_ in catalysis, sensors and energy applications. Continuing with the recent work of Uwihoreye et al.^32^, where they investigated the structural, electronic and optical properties of Ta:SnO_2_ thin films, they found that at low Ta concentrations, the lattice parameters of SnO_2_ are slightly reduced with the volume remaining almost unchanged. At low concentrations the incorporation of Ta atoms takes place in Sn sites and due to the smaller radius of Ta atoms (0.064 nm) compared to Sn atoms (0.065 nm) this leads to relative decrease of the lattice parameters. As they report, at higher concentration this phenomenon is reversed and the volume is increased. We believe that this is happening because at higher concentrations it is more likely for interstitials to form, and as we show, Ta_i_:SnO_2_ exhibits a higher volume than Ta_Sn_:SnO_2_. While our work predicts similar trends with the above mentioned experimental works, in order to explain completely and with great accuracy the above mentioned experimental results, different doping concentration should be examined, which is beyond of the scope of this paper. Ali and Islam^33^ investigated using DFT the effect of Ta doping in SnO_2_ and they also predicted that incorporation of Ta in SnO_2_ increases the volume of the supercell.

**Table 1:** Lattice parameters of Ta and V doped SnO_2_.

	a(Å)	c(Å)	Vol (Å^3^)
SnO_2_	4.71	3.18	70.95
V_Sn_:SnO_2_	4.69	3.18	69.95
V_i_:SnO_2_	4.89	3.21	76.76
Ta_Sn_:SnO_2_	4.72	3.19	71.07
Ta_i_:SnO_2_	4.94	3.20	78.09

**Figure 2:**
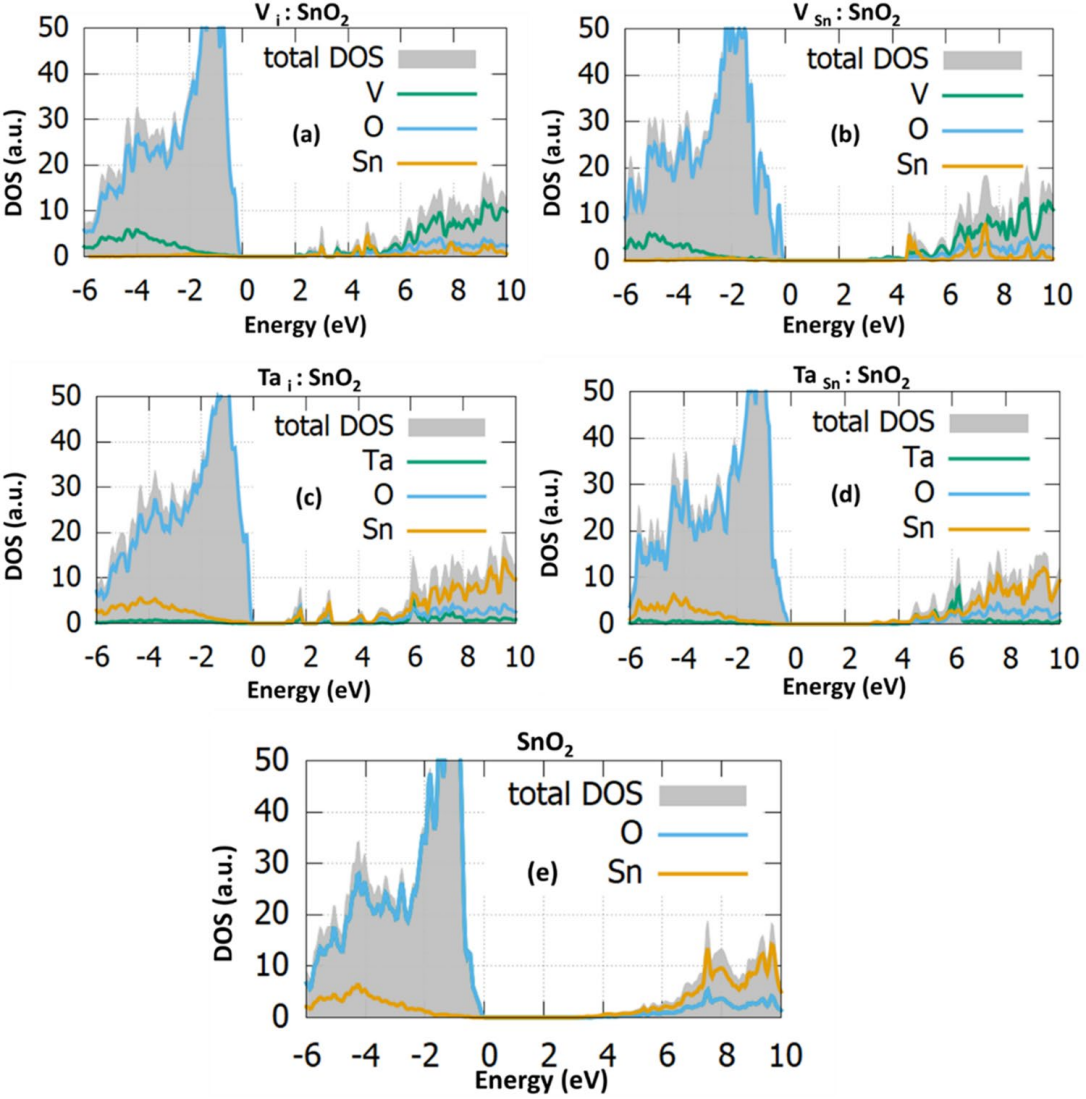
The total DOS and the projected density of states (PDOS) of (**a**) V_i_:SnO_2_, (**b**) V_Sn_:SnO_2_, (**c**) Ta_i_:SnO_2_ (**d**) Ta_Sn_:SnO_2_, and (**e**) undoped SnO_2_.

Continuing our work with the electronic investigation of Ta/V doped SnO_2_ we report in Fig. 2 the calculated total DOS and the (partial) PDOS for each doping case. As it is shown in Fig. 2e, the hybrid functional DFT calculations using PBE0 result in a bandgap value of 3.35 eV^13^, in excellent agreement with the experimentally determined bandgap^23^. Doping with Ta_i_ or V_i_ will result in a small increase of the band gap, however Ta and V at substitutional sites reduce the band gap by about 0.5 eV (refer to Table 2). Overall, The decrease of the band gap for the V and Ta doping, is caused by the overlapping of V-3d (Ta-3d) with O-2p. Those overlapping could initiate the formation not only of states near the band edges but also of intermediate bands (deep states). The increase of the bandgap in Ta_i_ could be attributed to the effect of electron doping which is responsible for shifting the fermi level into the conduction band, this phenomenon is also called Burstein-Moss effect^34^.

**Table 2:** The predicted electronic and optical constants.

	Bandgap	Refractive index	Reflectivity
SnO_2_	3.35	1.40	0.03
V_i_:SnO_2_	3.39	1.50	0.35
V_Sn_:SnO_2_	2.86	2.25	0.15
Ta_i_:SnO_2_	3.48	2.10	0.12
Ta_Sn_:SnO_2_	2.84	1.60	0.05

For both V_i_:SnO_2_ (Fig. 2a) and Ta_i_:SnO_2_ (Fig. 2c) in gap states form to the conduction band edge and the bandgap calculated to increase to 3.39 eV and 3.48 eV, respectively (Table 2). This formation of energy states in the middle of the bandgap can be advantageous for photocatalytic applications, however it is detrimental for photovoltaics and light emission diodes as they act as traps that reduce the device photocurrent and photogenerated charge carriers. Conversely, for V_Sn_:SnO_2_ (Fig. 2b) and Ta_Sn_:SnO_2_ (Fig. 2d) the band gap is reduced to 2.86 eV and 2.84 eV, respectively (refer to Table 1). This band gap reduction is attractive for photocatalytic applications.

**Figure 3:**
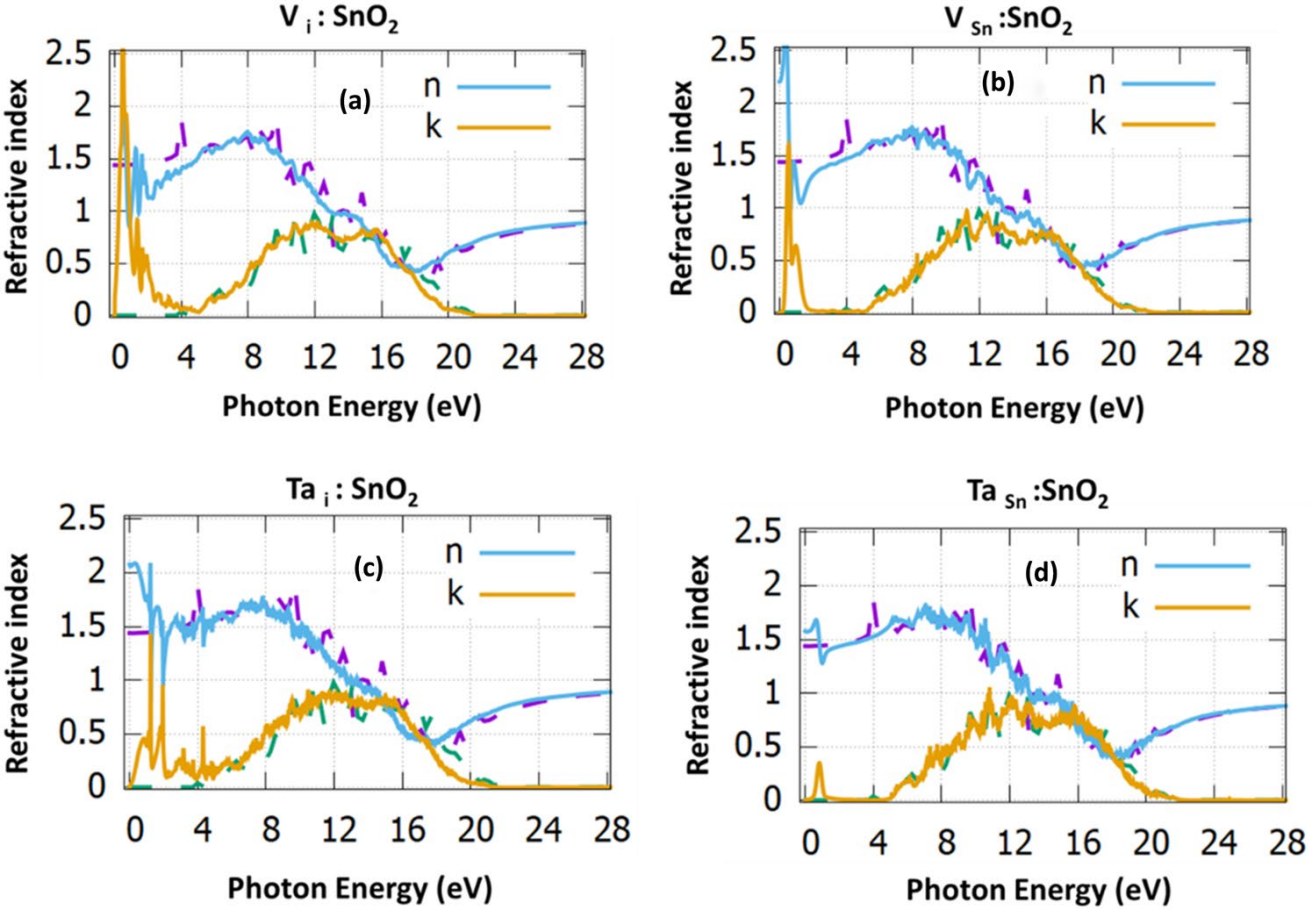
The refractive index for (**a**) V_i_:SnO_2_, (**b**) V_Sn_:SnO_2_, (**c**) Ta_i_:SnO_2_ and (**d**) Ta_Sn_:SnO_2_. The dotted purple and dotted green, correspond to the dielectric function of the undoped SnO_2_.

Figure 3 reports the refractive index with respect the phonon energy for all the doping cases considered. For zero frequency the refractive index is predicted to be a 1.40, in excellent agreement with previous theoretical studies but lower as compared to the experimental value (1.70)^35,36^. From Table 2 and Fig. 3 it is clear that there is an increase for the lower photon energies and a decrease in the upper energies.

**Figure 4:**
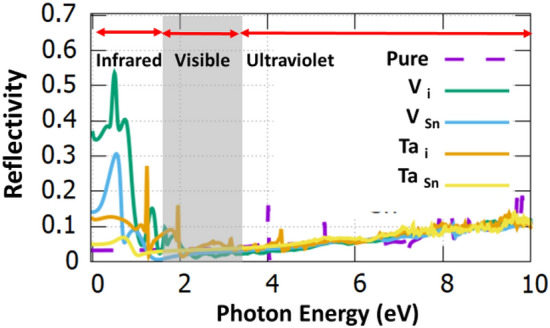
The reflectivity of the doped structures. The dotted purple line corresponds to undoped SnO_2_.

Figure 4 reports the reflectivity (i.e. amount of photons that are reflected) and it is predicted that V_i_:SnO_2_ has the highest reflectivity in the near-infrared region (refer to Table 2). Ta-doped SnO_2_ has low reflectivity in the infrared and visible region therefore it can be used as an antireflective coating.

**Figure 5:**
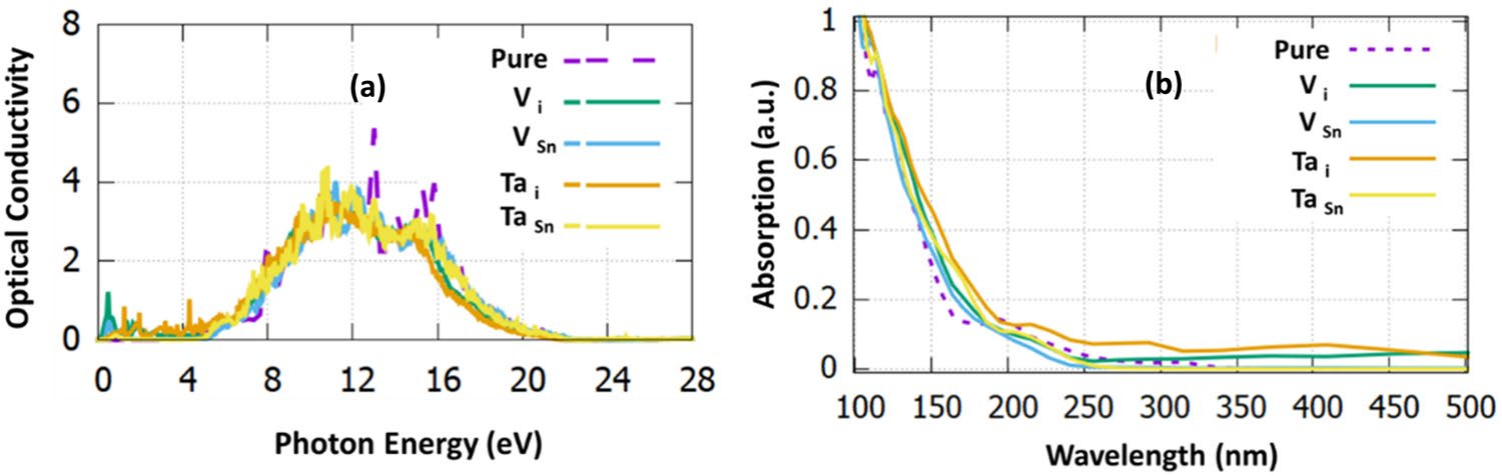
(**a**) The optical conductivity with respect to the photon energy and (**b**) the absorption coefficient with respect to the wavelength for the investigated dopants.

In Fig. 5a,b present the optical conductivity and absorption coefficient for all the doping cases considered here. The optical conductivity is effectively represented by the mobility of excitons (electron–hole pairs) a crucial parameter in the design of optical detectors^37^. The excitons are generated when photons have higher energy than the optical bandgap and because of the electronic charge neutrality do not contribute to the electrical conductivity^38^. The absorption of undoped SnO_2_ starts at 380 nm in fair agreement with the experimental value (400 nm)^39^. From Fig. 5b it is observed that that Ta_i_ and V_i_ have the highest absorption in the visible region.

### Surface SnO_2_

The investigation of the most exposed surface, in this case the (110) surface^13^, is of crucial importance for the application of doped SnO_2_ to various technologies such as gas sensors^40^ and photocatalytic hydrogen production^41^. Specifically for the gas sensors the adsorption and desorption of oxygen and gas molecules occurs on the surfaces of the material while for the hydrogen production the most high-energetic surface will play the role of the active site in photocatalytic reactions. As it is seen from the available literature, the studies of the (110) SnO_2_ surface are significantly less compared to the studies of the bulk.

**Figure 6:**
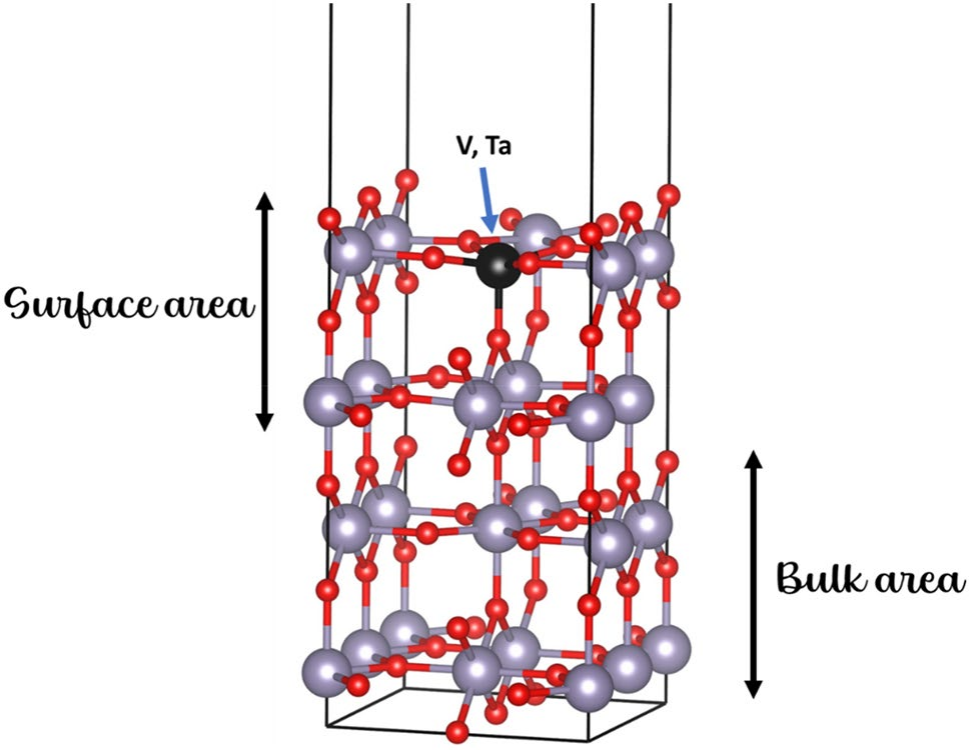
The slab model used for the V, Ta doped SnO_2_.

In this section, the electronic density of states changes of the undoped and the V. Ta doped SnO_2_ were investigated. The (110) surface is cleaved from the bulk SnO_2_ and it is represented with a nine-layer slab model, as it is presented in Fig. 6. The (110) surface consists of 16 SnO_2_ atoms and 32 O atoms while the size of the vacuum is chosen at 12 Å. For our calculations the bottom 4 layers are kept fixed during the geometry optimization in order to represent the bulk side while the top 5 are free to relax for the energy minimization. Our model has been used to various other studies, such as were the adsorption of hydrogen molecules on Cu-doped SnO_2_ surface is chosen^42^. Similarly, the same process was followed in this paper in order to simulate the minimum energy system for the doped surface. Furthermore, to accurate predict the DOS characteristics the hybrid functional PBE0 was used.

**Figure 7:**
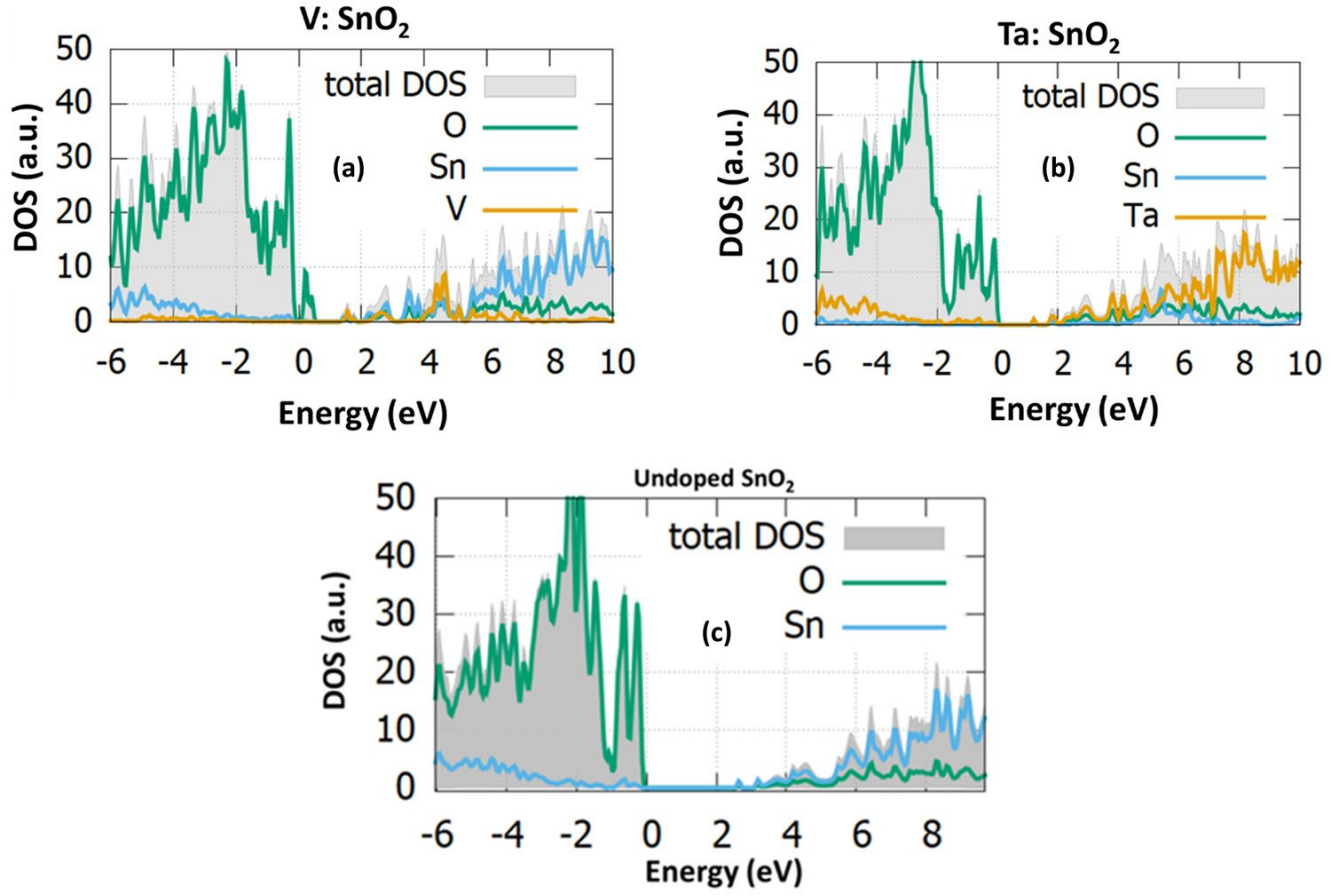
The total density of states (DOS) and the projected density of states (PDOS) of (**a**) V, (**b**) Ta and (**c**) undoped (110) SnO_2_ facet.

The total Density of states of the undoped and the V,Ta-doped SnO_2_ surface, as well as the partial density of states of O, Sn, V and Ta atom as are depicted in Fig. 7a–c. From our results in Fig. 7a it can be seen that V doping produces a surface band gap of nearly 2 eV. Compared to the pure SnO_2_ (110) facet it can be observed that the valence band is increased after V doping as some additional states are created at 0.5 eV. Our analysis shows that V doping reduces the bandgap of approximately 0.5 eV. Moreover, additional states are created at the conduction band edge. Continuing with the Ta doping, it can be observed that the bandgap is reduced to a value equal to 1.8 eV while the only energy states that arise are located at 1 eV. Compared to the undoped, Ta doping reduces the bandgap nearly 0.7 eV.

From the surface simulations it can be concluded that gap states that serve as electron traps are formed near the conduction band in all the doping cases. As these trap states are of great importance for the gas sensors and photocatalysis and so further experimental investigation is suggested.

## Conclusions

In the present DFT investigation the structural, electronic and optical properties of V and Ta doped SnO_2_ were calculated. Our first principles studies investigated the potential of these doping techniques for energy and sensing applications as it involved advanced hybrid calculations both for the bulk and the surface of SnO_2_. The DOS calculations revealed that there is a small bandgap increase for Ta_i_ and V_i_ doping, whereas for both the Ta and V substitutionals, the bandgap is decreased. Our calculations for the bulk agree well with other experimental reports and explain the trends that could be seen in them. The reduction of the band gap in the substitutional cases and the mid-gap states for the interstitial cases can be beneficial for photocatalytic applications while when the band gap is increased especially in the V_i_ case this can be beneficial for other applications such as electron transport layers. Furthermore, surface calculations indicate that these systems can be applicable for gas sensors as they can provide active sites for the sensing reactions to take place and also the gap states formed can further enhance these reactions. Therefore experimental work is necessary.

## Data Availability

The datasets used and/or analysed during the current study available from the corresponding author on reasonable request.
